# Eight Weeks of Resistance Training Is Not a Sufficient Stimulus to Improve Body Composition in Post-COVID-19 Elderly Adults

**DOI:** 10.3390/jcm14010174

**Published:** 2024-12-31

**Authors:** Katarzyna Kaczmarczyk, Kamila Płoszczyca, Karol Jaskulski, Miłosz Czuba

**Affiliations:** 1 Faculty of Rehabilitation, Józef Piłsudski University of Physical Education in Warsaw, Marymoncka 34, 00-968 Warsaw, Poland; katarzyna.kaczmarczyk@awf.edu.pl (K.K.); karol.jaskulski@awf.edu.pl (K.J.); 2 Department of Applied and Clinical Physiology, Collegium Medicum University of Zielona Gora, Licealna 9, 65-417 Zielona Góra, Poland; milosz.czuba@awf.edu.pl

**Keywords:** resistance training, physical activity, COVID-19, older adults, body composition, BIA, DXA

## Abstract

**Background**: This study sought to assess how body mass (BM) and body composition in post-COVID-19 elderly adults were affected by 8 weeks of resistance training. An additional goal was to determine the agreement between Bioelectrical Impedance Analysis (BIA) and Dual Energy X-Ray Absorptiometry (DXA) in elderly people. **Methods**: Participants were randomly assigned to an intervention Group, which engaged in 8 weeks of resistance training, and a Control Group, which was advised to maintain their usual activity levels. Before and after the intervention, the body composition was analyzed via the BIA and DXA methods. **Results**: We found no statistically significant changes in BM or body composition following resistance training. BIA was found to overestimate the participants’ baseline BM and fat-free mass (FFM) and to underestimate the fat mass (FM), compared to the DXA method. There were no significant differences in intervention-induced changes in FM and FFM measured by BIA and DXA. **Conclusions**: Moderate intensity resistance training lasting 8 weeks was not found to be a sufficient stimulus to improve BM and body composition in post-COVID-19 elderly adults. We also conclude that BIA may serve as a viable alternative to DXA for measuring longitudinal changes in body composition in elderly people.

## 1. Introduction

COVID-19 disease, caused by the SARS-CoV-2 virus, has affected millions of people worldwide [1]. Currently, scientific research is increasingly focused on analyzing persistent or new symptoms in people who have recovered from COVID-19 [2]. Persistent symptoms and/or delayed or long-term complications that persist or begin more than four weeks after the onset of symptoms of SARS-CoV-2 infection have been termed Long COVID, Post-Acute COVID-19 Syndrome, or Post-Acute Sequelae of SARS-CoV-2 infection [3,4]. Long COVID covers a diverse spectrum of symptoms that impact everyday activities and quality of life, including persistent fatigue, dyspnea, cognitive deficits, musculoskeletal discomfort, impaired mobility, and gastrointestinal upset [3,5]. Most of these symptoms lead to limitations in patients’ capacity for physical activity and, as a consequence, may cause unfavorable changes in body mass and body composition.

Body mass index (BMI) and body composition have been shown to be factors influencing the severity of COVID-19 and persistence of long-term COVID-19 symptoms [6]. Excess fat tissue promotes excessive inflammation at the systemic level and reduces the immune response, potentially affecting the outcome of COVID-19 [7]. Additionally, individuals with low muscle mass and excess body fat, especially visceral fat, may be at increased risk of developing long-term symptoms of COVID-19, which may persist for longer after infection and slow recovery [8,9]. It has been established that individuals hospitalized with COVID-19 are at increased risk of muscle loss and developing acute sarcopenia [10]. The long-term effects of COVID-19 on the musculoskeletal system may be more pronounced in older people, due to their generally low levels of physical activity, which were further reduced during the quarantine period of COVID-19 [11].

Evidence suggests that physical activity plays a significant role in treating and resolving long-term symptoms of COVID-19 [12]. In general, a long-term physical exercise program improves the physical fitness and quality of life in elderly people [13,14]. It has been suggested that structured physical activity should be an integral part of the rehabilitation for individuals experiencing persistent effects of the virus [12,15].

Resistance training is recommended to delay and mitigate the negative effects of sarcopenia [16]. It has been shown to be an effective strategy for improving physical fitness, muscle strength, and body composition, which translates into improved quality of life in elderly adults [16]. Resistance training is one of the elements of the proposed rehabilitation interventions in long COVID-19 [10,17]. Previous work has revealed that resistance training, either alone or in combination with aerobic training, is effective in increasing skeletal muscle mass and muscle strength, as well as improving muscle activation and postural stability in individuals with long COVID-19 [18,19,20,21,22,23,24]. However, the isolated effect of resistance training on body mass and composition in the post-COVID-19 elderly population is unknown. Therefore, the primary purpose of our study was to assess the changes in body mass and body composition of post-COVID-19 elderly adults who participated in an 8-week Resistance Training protocol.

Monitoring body mass and body composition seems to be one of the important elements of controlling the rehabilitation process of post-COVID-19 elderly individuals. In epidemiological and clinical conditions, Bioelectrical Impedance Analysis (BIA) and Dual Energy X-Ray Absorptiometry (DXA) are widely used methods to assess body composition. DXA provides accurate estimates of bone mineral, fat, and lean soft tissue, and it is currently considered the reference technique in clinical practice [25,26]. On the other hand, BIA has gained wide acceptance, mainly as a field technique, due to its speed and ease of measurement, compact size, and affordability. BIA measures the electrical properties of body tissues and estimates body composition parameters. However, changes in fat-free mass hydration that occur with aging and disease states may cause errors in the assessment of body composition using the BIA method in elderly adults [25,27,28,29]. Therefore, an additional goal of our study was to determine the degree of agreement between BIA and DXA, both with respect to baseline measurements, and the magnitude of body composition changes in post-COVID-19 elderly people.

## 2. Materials and Methods

### 2.1. Participants

Participants in this study were drawn from residential care homes, primary healthcare centers, and educational programs for seniors (University of the Third Age). To qualify for inclusion, individuals had to be 65 years of age or older and must have tested positive for SARS-CoV-2 on an RT-PCR test or antibody test, performed within the 3 to 12 months before the start of the study. Additionally, participants were required to report at least one lingering symptom associated with post-COVID-19, including fatigue, muscle weakness, dizziness, headaches, cognitive impairments, reduced exercise tolerance, or depressive symptoms. Both male and female participants were included. Exclusion criteria included being under the age of 65, having an existing cardiovascular condition with prolonged oxygen saturation levels below 95% lasting over one minute, autonomic nervous system disorders such as orthostatic intolerance, or severe illnesses like cancer.

Among the participants, 92% had received at least one dose of the anti-SARS-CoV-2 vaccine, and only 27% had contracted COVID-19 before vaccination. The average time since disease onset for those meeting the inclusion criteria was 9 months. Of these participants, 33% reported a mild infection, 51% moderate, 10% severe, and 6% very severe. A post-infection interview assessed symptoms, with 54.9% reporting dizziness and equilibrium disorders, 35.3% experiencing perceived muscle weakness, 31.4% reporting exercise intolerance, and 19.6% experiencing memory and concentration issues. Less frequent symptoms included cough, dyspnea, libido deterioration, insomnia, and loss of taste and smell (Table 1).

**Figure 1:**
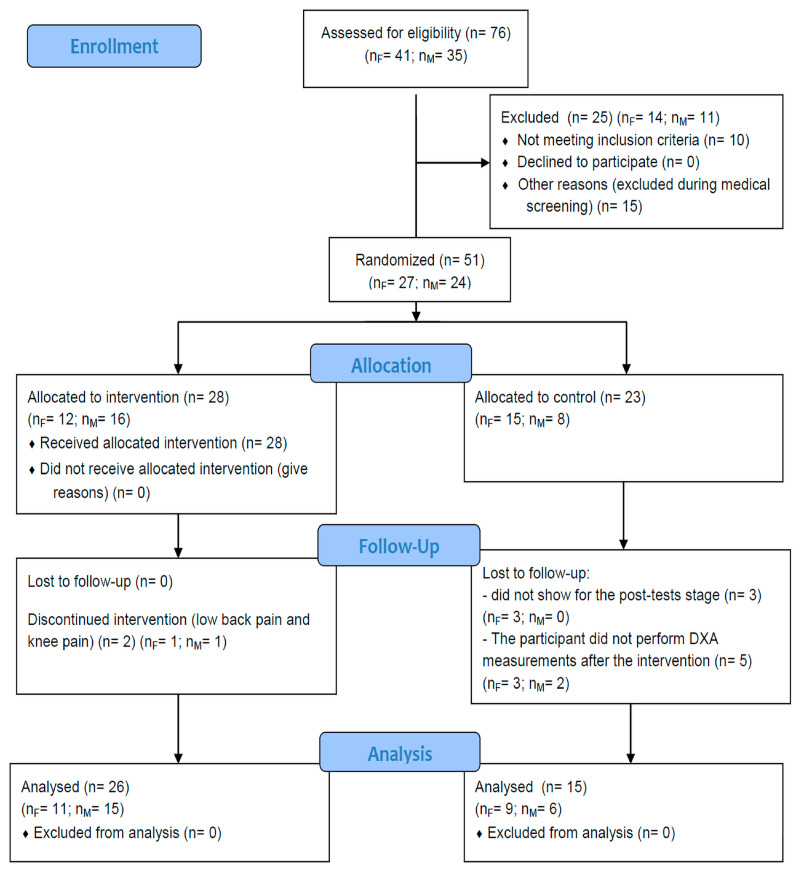
CONSORT flow diagram. F—females; M—males.

Those participants who satisfied the eligibility requirements underwent medical screening evaluations; those that passed were allocated into one of two groups through a randomized process using an Excel-based number generator. The first group, referred to as the Intervention Group, underwent a structured resistance training program, while the second group, labeled the Control Group, was instructed to continue with their routine physical activities without any modifications. The study took place at the Central Laboratory of the Józef Piłsudski University of Physical Education in Warsaw, Poland, in compliance with the Declaration of Helsinki and approved by the University’s Ethics Committee (SKE 01-41/2022, 21 December 2022). The study protocol was registered on clinicaltrials.gov (NCT05934279). The CONSORT diagram is presented in Figure 1.

The data from the 41 post-COVID-19 elderly adults were analyzed. Baseline anthropometric characteristics of the groups are shown in Table 2. The required minimum total number of subjects (*n* = 40) was determined using the G*Power program (Version 3.1.9.4), assuming detection of medium-sized effects (*η*^2^ = 0.06) at a significance level of *α* = 0.05 and statistical power of 0.85 [30].

### 2.2. Procedures

All participants of the study were informed about possible contraindications for performing body analysis and confirmed their absence. Both BIA and DXA measurements were performed on the same day, in the morning; all patients had a fasting period of at least 8 h. Additionally, participants were instructed not to use diuretics for 7 days before the measurements, not to consume alcohol, and to avoid physical exercise for at least 12 h before the study. All participants were asked to empty their bladders before starting the measurements and to remove all metal objects.

### 2.3. Bioelectrical Impedance Analysis

Body composition analysis was performed using the Jawon Medical IOI-353 analyzer (Yuseong, Republic of Korea), certified by EC0197 and compliant with the MDD 93/42EEC Directive. The analysis followed the device’s manual and utilized bioelectrical impedance analysis (BIA), a reliable, safe, and non-invasive method using eight electrodes. The BodyPass 1.0 software was employed to measure body mass (BM) [kg], fat mass (FM) [kg], percentage of body fat [%FAT], fat-free mass (FFM) (kg), soft lean mass [kg], total body water (TBW) [%], and BMI [kg/m^2^]. Participants were informed about contraindications and pacemakers and confirmed their absence. The study was conducted in the morning after participants had fasted for at least eight hours and rested. Prior to the examination, participants underwent an interview, removed their socks, metal objects, and jewelry, and then proceeded with the body composition analysis in a standing position.

### 2.4. Dual-Energy X-Ray Absorptiometry

Whole-body composition assessments were carried out utilizing the Lunar Prodigy Pro DXA system (GE Healthcare, Madison, WI, USA). The scans were processed using GE Encore v16 software under standardized conditions to ensure consistency. Measurements took place in the morning hours, following an overnight fast. Participants were dressed only in light undergarments, free of any metal accessories, and were asked to empty their bladders prior to the procedure.

The DXA machine was calibrated daily before examinations using a quality assurance calibration block. The assessed whole-body composition parameters included: body mass (BM) [kg], fat mass (FM) [kg], percentage of body fat [%FAT], fat-free mass (FFM) [kg], lean soft tissue [kg], bone mass [kg], and BMI [kg/m^2^]. Subjects were positioned on the scanner in accordance with the operator’s manual.

### 2.5. Training Intervention

Resistance training sessions were held twice weekly, lasting 60 min each, over a period of eight weeks, in line with the guidelines set forth by World Physiotherapy [31]. The training regimen was designed by a multidisciplinary team comprising physicians, physiotherapists, and certified strength training coaches, as outlined in Kaczmarczyk et al. [18]. The program targeted an intensity level of 70% of one repetition maximum (1RM) and included three sets of 12 repetitions for each exercise: incline bench press, 45-degree leg press, lat pulldown, trunk crunch, T-bar row, leg extension, and leg curl. Rest intervals of two minutes were incorporated between sets to allow for passive recovery. Training loads were adjusted incrementally by 5 kg whenever a participant successfully completed all repetitions for a given exercise. Prior to each session, participants underwent health screenings, including measurements of heart rate, blood pressure, and oxygen saturation. Those with blood pressure readings exceeding 160/100 mmHg or heart rates outside the range of 50–100 beats per minute were excluded from that day’s session.

During the experiments, the participants consumed a mixed diet (50% CHO, 30% Fat, 20% Pro). Daily energy intake was set at an average of 1597 ± 122 kcal in women and 2079 ± 285 kcal in men. Total energy expenditure was estimated based on basal metabolic rate and physical activity level index (PAL = 1.5; low activity). During the initial screening, the composition and caloric value of the diet for each participant were determined, and nutritional recommendations were provided. Participants declared that they maintained the recommended diet throughout the experiment.

### 2.6. Statistical Analysis

Statistical analysis was performed using STATISTICA 14.0 (TIBCO Software Inc., Palo Alto, CA, USA 2020). The results were presented as arithmetic means (*x*) ± standard deviations (SD). The statistical significance was set at *p* < 0.05. Prior to all statistical analyses, normality of the distribution of variables was checked using the Shapiro–Wilk test. Analysis of variance (ANOVA) for repeated measures (GROUP × TESTING SESSION) was used to determine the differences in each of the dependent variables. When significant differences were found, the post-hoc Tukey test was used. The Wilcoxon test was used to assess differences between the magnitude of changes (delta) in variables that were recorded under the influence of the intervention. The agreement of DXA and BIA results for body mass and body composition was assessed using the Bland–Altman method. Linear correlations (Pearson’s *r*) were also performed to test the relationship between variables predicted by BIA and DXA, as well as the relationship between post-infection symptoms and body composition indices.

## 3. Results

There were no statistically significant changes in body mass (BM) and body composition measured by DXA and BIA in both Intervention and Control groups (Table 3 and Table 4). No correlation was found between disease outcome (post-infection symptoms reported by participants) and body mass or body composition (Table 5).

The correlation coefficients (*r*) of baseline BM, fat mass (FM), and fat-free mass (FFM) between DXA and BIA were strong (BM: *r* = 0.99; FM: *r* = 0.96; FFM: *r* = 0.97) with *p* < 0.001.

**Figure 2:**
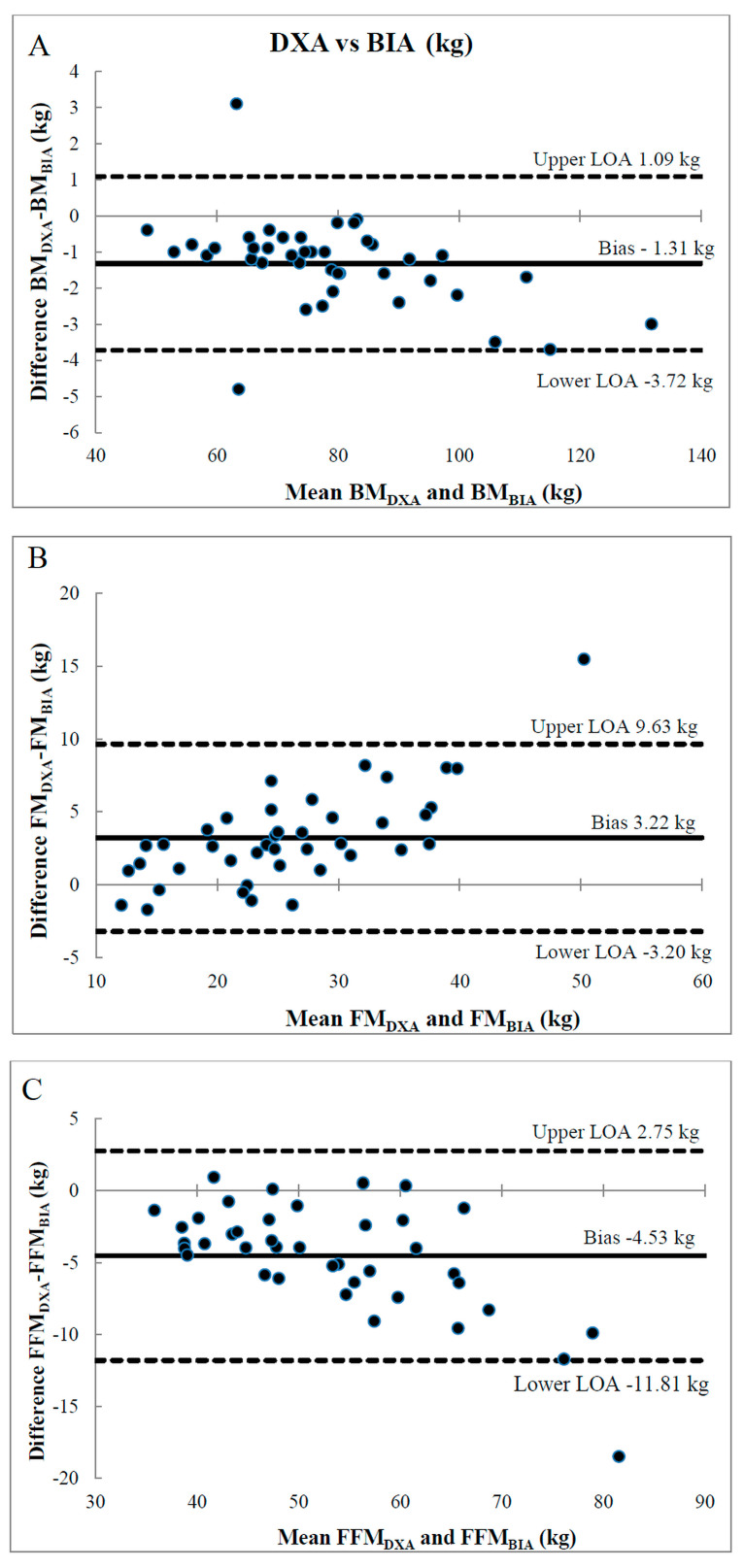
Bland–Altman plots comparing body mass (BM) (**A**), fat mass (FM) (**B**), and fat-free mass (FFM) (**C**) determined by DXA and BIA methods at baseline. The solid line represents the bias, and the dashed lines represent upper and lower limits of agreement (LOA = Bias ± 1.96 × SD). BIA—Bioelectrical Impedance Analysis; DXA—Dual Energy X-Ray Absorptiometry.

The Bland–Altman plot for comparing the two methods in the assessment of BM and body composition showed that the BIA overestimated the participants’ baseline BM by −1.31 ± 1.23 kg (Upper LOA = 1.09 kg; Lower LOA = −3.72 kg), underestimated the FM by 3.22 ± 3.27 kg (Upper LOA = 9.63 kg; Lower LOA = −3.20), and overestimated FFM by −4.53 ± 3.72 kg (Upper LOA = 2.75 kg; Lower LOA = −11.81 kg) as compared to the DXA method (Figure 2). The participants’ baseline FM correlated with the underestimation of FM (*r* = 0.79, *p* < 0.001) and overestimation of FFM by BIA (*r* = −0.80, *p* < 0.001).

The magnitude of intervention-induced BM changes differed significantly between BIA and DXA measurements. BIA recorded a significantly greater difference in BM pre- and post-experiment than DXA in the Intervention Group (*p* < 0.01). There were no significant differences for FM and FFM changes measured by the two methods (Table 6).

## 4. Discussion

Resistance training has previously been shown to be the most effective intervention for increasing muscle strength and growth and reducing body fat percentage in elderly adults [16,32]. In our previous research, we showed that muscle strength and function utilization in post-COVID survivors improved after applying the proposed resistance training protocol [18]. In our current study, we did not detect significant alterations in body mass and body composition after an 8-week resistance training protocol in elderly adults post-COVID-19 infection, regardless of the measurement method used (DXA or BIA).

Previous research has demonstrated that a six-week multidisciplinary rehabilitation program combining physical activity, therapeutic education, and psychotherapy sessions can result in reductions in abdominal fat mass, waist circumference, and cellular hydration while simultaneously promoting gains in skeletal muscle mass among 60-year-old individuals recovering from long COVID-19 [19].

Physical training as part of the rehabilitation program consisted of aerobic, resistance, and breathing exercises and was carried out for 90 min, 3 times a week [19]. In turn, Sordi et al. [33] reported that 8 weeks of multi-professional intervention, including aerobic and resistance exercises, did not produce significant improvements in body composition in overweight COVID-19 survivors. Similar conclusions were reached by Jakše et al. [34] after applying an eight-week swimming program. They observed no significant body mass loss or body composition improvements in post-COVID-19 patients with an average age of 59 ± 14 years.

Training procedures implemented during rehabilitation in post-COVID-19 patients are characterized by different training types, variable intensity, volume, and exercise selection [19,24,33,34], which limits the interpretability of research results. It also happens that the details of the training program are missing [19]. Currently, it is difficult to determine whether several weeks of isolated resistance training are effective in improving body mass and composition and which element of training has a key impact on its effectiveness in post-COVID-19 elderly people. We suspect that the lack of improvement in body composition in our study may have been due to the insufficient training frequency (twice a week for 60 min) and the length of the training program (8 weeks). It has been suggested that a longer duration of resistance training program is more effective than a shorter duration in improving muscle mass, body fat mass, and muscle strength in elderly adults, and the duration of training intervention should be at least 12 weeks [35,36].

Another important factor influencing the effectiveness of resistance training is a proper diet [37]. The combination of diet and resistance training in elderly people can result in an improvement in body composition, especially an increase in muscle mass [38]. Furthermore, a well-balanced diet is considered an integral element of a comprehensive strategy to minimize the effects of COVID-19 and promote faster recovery [34]. Especially in elderly people, the lack of physical activity caused by COVID-19 lockdown may have had a negative impact on nutrition behavior, leading to insufficient intake of nutrients necessary to maintain muscle mass and an increase in sarcopenic obesity [39]. The mixed diet we used was insufficient to improve body composition in our subjects over an 8-week period. It is possible that increasing protein intake would enhance the effectiveness of resistance training, as previously demonstrated in studies of older adults [37,40,41]. This aspect is worth considering in future research.

The second aim of our study was to assess the agreement between BIA and DXA, both in relation to baseline measurements, and to assess changes in body composition in elderly people. DXA is a method that often serves as a reference standard when assessing body composition [25]. It is recognized that there is no reason to specifically challenge the validity of DXA body composition measurements in healthy elderly populations [42]. Although DXA is becoming more available, its use also presents some challenges, such as the cost of DXA, logistical constraints in administering the test (time and space requirements), and the involvement of trained personnel [43]. Alternatively, BIA is widely used in clinical and research practice, and its advantages include affordability, compact size, shorter measurement time, and ease of use compared to DXA [27].

It has been suggested that body composition analysis using BIA should be routinely included in elderly people in order to early detect sarcopenia, obesity, or abnormal hydration levels and implement interventions aimed at improving the quality of life and health of elderly people [44]. This seems particularly important in the case of elderly people, in whom long COVID could disturb the functioning of many body systems, including increasing the risk of sarcopenia due to inflammation, malnutrition, and muscle catabolism [45,46].

Previous research findings support a strong correlation coefficient (*r* > 0.90) between BIA and DXA estimates of body composition [26,43,47,48,49]. In line with the previous reports, the present study observed strong correlations in FM and FFM measurement between DXA and BIA methods (*r* > 0.96). However, despite the strong correlation, the degree of agreement reported between DXA and BIA varies [26,43,47,48,49]. It has been noted that BIA-DXA agreement is poorer in individuals with higher BMI, in whom BIA underestimates FM and overestimates FFM as compared to DXA [26,43,49]. Estimation of body composition by BIA may also be inaccurate in elderly people [42,50].

BIA is based on the measurement of bioimpedance, which results from the conductive properties of the human body and its response to the passage of sinusoidal current through its various biological components. The current flows through the body at different rates depending on the tissue it passes through. Muscle tissue is composed essentially of water and electrolytes and is a good electrical conductor, while cell membranes, body fat, and bones are composed of anhydrous materials with reduced conductivity [28,29,51]. One of the basic assumptions of BIA is that FFM is calculated assuming a hydration fraction for FFM, typically 0.73, including intracellular fluid (ICF) and extracellular fluid (ECF) [52]. However, the ECF/ICF ratio is highly variable in individuals with higher BMI. The inconsistent expansion in ECF and ICF as adipose tissue mass increases will increase the estimated percentage of total body water (TBW), which may cause the BIA to overestimate FFM and consequently underestimate FM [53,54]. Moreover, in elderly people, age-related changes related to demineralization of bone mass, muscle mass depletion, and changes in the hydration of the FFM (decreased FFM density) should be taken into account. These factors, alone or together with improper body hydration, may lead to large errors in the assessment of body composition using the BIA method [42,50].

In our study, for elderly participants with mean BMI 28.0 ± 5.1 kg/m^2^, the BIA underestimated FM and overestimated FFM compared with DXA on average by 3.22 kg and 4.53 kg, respectively. Additionally, our results showed that the higher the FM, the greater the underestimation of FM, and the overestimation of FFM by BIA was observed. The results of the current study are comparable to previous reports. Ellison et al. [43] showed that in adults (61.4 ± 6.9 years) with mean BMI 38.6 ± 5.0 kg/m^2^, the underestimation of FM and overestimation of FFM by BIA were on average 4.5 kg and 4.6 kg, respectively. Achamrah et al. [49] demonstrated that for individuals with BMI > 18.5 and <40.0, BIA overestimated FFM from 3.38 to 8.28 kg and underestimated FM from 2.51 to 5.67 kg compared with the DXA method, while people with BMI < 18.5 body composition values were very close (difference < 1 kg).

High LOAs values reported in several BIA/DXA comparison studies indicate that association between BIA and DXA methods at the individual level is lacking [43,48,49,55]. In our current study, baseline LOAs were −3.20 to 9.63 kg for FM and −11.81 to 2.75 kg for FFM. This means that 95% of the differences in FM and FFM measured by BIA and DXA can be expected to fall within these ranges. The reported LOAs for FM and FFM exceed 39% and 23%, respectively, which are considered large and indicate a high degree of potential clinical error [43,52]. However, our results also revealed that the magnitudes of changes (delta) in FM and FFM that were recorded under the influence of the 8-week intervention did not differ significantly between the DXA and BIA methods. Similarly, in a study conducted by Ellison et al. [43], mean FM and FFM changes over the course of a 16-week weight loss program were almost identical for DXA and BIA. Kyle et al. [29] found that observing longitudinal changes in body composition using BIA is practically possible in people with a BMI of 16–34 kg/m^2^ without abnormal hydration. Our results confirm the above findings. Despite poor agreement between BIA and DXA measurements for baseline FM and FFM values, the magnitude of changes recorded after the 8-week intervention did not differ between the methods.

### Limitations and Future Perspectives

Our study had a number of limitations that should be addressed. The first is the lack of full control over the participants’ diet. While the subjects were given dietary guidelines, greater control of their diet, for example, accommodation of participants in one place and implementation of a nutritional program for the duration of the experiment, could allow for a more precise determination of the impact of resistance training on body composition. A second limitation is the absence of biochemical laboratory data for participants, as well as the inability to consistently monitor symptom progression in both the control and intervention groups. In future research, we aim to address these gaps by incorporating larger sample sizes and evaluating changes across a broader spectrum of symptoms.

A further limitation is that we did not include an additional control group—individuals who did not have COVID-19 infection or had no further sequelae. This would allow us to determine whether the effects of resistance training were related to a specific cohort (elderly adults with long COVID-19) or whether they applied more broadly to the elderly population, regardless of the occurrence of post-COVID-19 symptoms.

Physical activity appears to play a significant role in the management and resolution of long-term COVID-19 symptoms. Therefore, it is important to establish which types of exercises (e.g., aerobic, resistance) and which training program (e.g., training frequency, intensity, volume) are the most effective in improving health and quality of life in people post-COVID-19 infection. For example, future studies should include comparisons of resistance training at different intensities or volumes to more precisely define practice guidelines for post-COVID patients.

## 5. Conclusions

Based on our results, we conclude that moderate-intensity resistance training lasting 8 weeks (2×/week; 60 min per session) is not a sufficient stimulus for the improvement of body mass and body composition in post-COVID-19 elderly adults. The results of this study also show that BIA underestimates baseline FM and overestimates FFM compared to DXA in elderly adults and, to this extent, should be used with caution in clinical and research practice. However, FM and FFM changes recorded after 8 weeks of intervention did not differ between measurement methods, thus we conclude that BIA may serve as a viable alternative to DXA for measuring the longitudinal changes in body composition in elderly people.

## Figures and Tables

**Table 1:** Post-infection symptoms in recruited participants.

Symptoms	Female (*n* = 27)		Male (*n* = 24)		Total (*n* = 51)	
	*n*	%	*n*	%	*n*	%
Dizziness and equilibrium disorders	20	74.1	8	33.3	28	54.9
Muscle weakness	13	48.1	5	20.8	18	35.3
Exercise intolerance	9	33.3	7	29.2	16	31.4
Memory and concentration issues	7	25.9	3	12.5	10	19.6
Cough	5	18.5	3	12.5	8	15.7
Loss of taste and smell	4	14.8	2	8.3	6	11.8
Dyspnea	1	3.7	1	4.2	2	3.9
Insomnia	2	7.4	0	0	2	3.9
Libido deterioration	0	0	1	4.2	1	2.0

**Table 2:** Anthropometric characteristics of the tested groups at baseline (Mean ± SD).

	Intervention (F = 11, M = 15)	Control (F = 9, M = 6)	Student’s *t* test	
			*t*	*p*
Female				
Age (years)	69.3 ± 5.2	73.0 ± 8.2	−1.24	0.2312
Body mass (kg)	65.3 ± 10.5	75.9 ± 12.1 *	−2.12	0.0484
Height (m)	163.1 ± 7.6	161.8 ± 6.2	0.42	0.6814
BMI (kg/m^2^)	24.7 ± 4.9	29.1 ± 3.3 *	−2.26	0.0361
Male				
Age (years)	69.5 ± 4.8	76.2 ± 7.0 *	−2.53	0.0205
Body mass (kg)	87.7 ± 15.1	90.9 ± 23.2	−0.37	0.7128
Height (m)	176.7 ± 6.9	179.7 ± 5.0	−0.97	0.3448
BMI (kg/m^2^)	28.1 ± 3.5	27.9 ± 5.9	0.04	0.9692
Total				
Age (years)	69.4 ± 4.9	74.3 ± 7.6 *	−2.50	0.0167
Body mass (kg)	78.2 ± 17.3	81.9 ± 18.2	−0.65	0.5182
Height (m)	170.9 ± 9.8	168.9 ± 10.6	0.61	0.5473
BMI (kg/m^2^)	26.6 ± 4.4	28.6 ± 4.4	−1.41	0.1676

* *p* < 0.05—compared to Intervention group; BMI—Body mass index.

**Table 3:** The body mass and body composition of the participants before and after the intervention measured by the DXA method (Mean ± SD).

DXA	Testing Session	Control (*n* = 15)	Intervention (*n* = 26)	ANOVA (Group × Testing Session)		
				*F*	*p*	*η* ^2^
Body mass (kg)	Before	80.60 ± 17.78	76.89 ± 16.69	0.06	0.8087	0.002
	After	80.65 ± 17.08	76.81 ± 16.74			
Fat Mass (kg)	Before	30.86 ± 11.15	25.60 ± 8.63	0.12	0.7262	0.003
	After	30.42 ± 10.53	25.31 ± 8.72			
%FAT	Before	37.72 ± 8.18	32.90 ± 6.95	0.12	0.7267	0.003
	After	37.26 ± 8.04	32.57 ± 7.28			
Fat Free Mass (kg)	Before	49.74 ± 10.05	51.29 ± 10.91	0.43	0.5177	0.012
	After	50.23 ± 10.17	51.50 ± 11.16			
Lean Soft Tissue (kg)	Before	47.19 ± 9.43	48.66 ± 10.40	0.57	0.4561	0.014
	After	47.70 ± 9.59	48.86 ± 10.65			
Bone Mass (kg)	Before	2.54 ± 0.66	2.63 ± 0.59	3.10	0.0861	0.074
	After	2.52 ± 0.64	2.64 ± 0.58			
BMI (kg/m^2^)	Before	28.76 ± 4.43	27.05 ± 4.58	0.04	0.8397	0.001
	After	28.57 ± 4.24	26.90 ± 4.51			

DXA—Dual Energy X-Ray Absorptiometry; %FAT—percentage of body fat; BMI—Body mass index.

**Table 4:** The body mass and body composition of the participants before and after the intervention measured by the BIA method (Mean ± SD).

BIA	Testing Session	Control (*n* = 15)	Intervention (*n* = 26)	ANOVA (Group × Testing Session)		
				*F*	*p*	*η* ^2^
Body mass (kg)	Before	81.93 ± 18.24	78.20 ± 17.30	0.25	0.6169	0.006
	After	81.17 ± 18.11	77.78 ± 17.22			
Fat Mass (kg)	Before	27.14 ± 8.22	22.67 ± 6.72	0.85	0.3614	0.021
	After	26.75 ± 7.63	21.79 ± 5.95			
%FAT	Before	33.11 ± 7.18	28.87 ± 5.70	0.49	0.4865	0.012
	After	32.74 ± 7.03	28.13 ± 5.43			
Fat Free Mass (kg)	Before	54.81 ± 13.35	55.51 ± 12.93	1.48	0.2312	0.037
	After	54.40 ± 13.55	56.01 ± 13.44			
Soft Lean Mass (kg)	Before	50.25 ± 12.36	51.20 ± 11.95	0.03	0.8713	0.001
	After	50.35 ± 12.51	51.39 ± 12.51			
Total Body Water (%)	Before	39.52 ± 9.59	40.08 ± 9.28	0.01	0.9383	0.000
	After	39.60 ± 9.67	40.20 ± 9.69			
BMI (kg/m^2^)	Before	28.63 ± 4.37	26.64 ± 4.36	1.24	0.2718	0.031
	After	28.61 ± 4.34	26.39 ± 4.31			

BIA—Bioelectrical Impedance Analysis; %FAT—percentage of body fat; BMI—Body mass index.

**Table 5:** Correlation coefficients between post-infection symptoms and body composition.

	*r*	*p*
Body mass × Symptoms	−0.0334	0.836
BMI × Symptoms	0.0134	0.934
Fat Mass × Symptoms	0.1649	0.303
FFM × Symptoms	−0.2082	0.191

Symptoms—Post-infection symptoms reported by participants: dizziness and equilibrium disorders, muscle weakness, exercise intolerance, memory and concentration issues, cough, dyspnea, libido deterioration, insomnia, and loss of taste and smell.

**Table 6:** The magnitude of changes (∆) in body mass, fat mass, and fat-free mass measured by DXA and BIA methods (Mean ± SD).

	Group	Method		Wilcoxon Test (Method Comparison, DXA vs. BIA)		
		DXA	BIA	*T*	*p*	*r*
∆Body mass (kg)	Control	0.05 ± 2.28	−0.81 ± 2.95	26.5	0.0571	0.491
	Intervention	−0.08 ± 1.16	−0.38 ± 1.39 **	59.0	0.0093	0.509
∆Fat Mass (kg)	Control	−0.43 ± 1.51	−0.41 ± 1.69	48.0	0.4955	0.176
	Intervention	−0.29 ± 1.05	−0.87 ± 1.59	110.0	0.0962	0.325
∆Fat Free Mass (kg)	Control	0.49 ± 1.57	−0.41 ± 2.89	43.0	0.3343	0.249
	Intervention	0.22 ± 1.08	0.50 ± 1.87	164.0	0.7702	0.057

** *p* < 0.01—compared to DXA method. BIA—Bioelectrical Impedance Analysis; DXA—Dual Energy X-Ray Absorptiometry.

## Data Availability

The datasets generated and analyzed during the current study are not publicly available due to the restrictions involved when obtaining ethical approval for our study, which commit us to share the data only with members of the research team but allow data to be made available from the corresponding author upon reasonable request.
